# A Discussion on "Treatment by Ionisation or Cataphoresis"
^1^Held at the meeting of the Bristol Medico-Chirurgical Society, May 12th, 1909.


**Published:** 1909-06

**Authors:** H. P. Tayler, H. Lewis Jones

**Affiliations:** Medical Officer in Charge of the Electrical Department in St. Bartholomew's Hospital, London


### A DISCUSSION[1] ON "TREATMENT BY IONISATION OR CATAPHORESIS."

OPENED BY

H. P. TAYLER, M.A., M.B. Cantab.,

AND

H. LEWIS JONES, M.A., M.D. Cantab., F.R.C.P.,

*Medical Officer in Charge of the Electrical Department in St. Bartholomew's Hospital, London.*

DR. H. P. TAYLER opened the discussion with the following remarks:—

At a recent clinical evening of the British Medical Association so much attention was drawn to a case of rodent ulcer which

1 Held at the meeting of the Bristol Medico-Chirurgical Society, May 12th, 1909.

had been treated by zinc ionisation that I thought it might be of interest if I related the history of several cases which I have treated by ionic medication, and gave an account of the technique of the treatment.

As my first case was one of rodent ulcer, perhaps I may best begin with these, and I will mention them in the order of their importance, rather than in the order of the dates of their occurrence.

**Case 1.**—A male, aged about 50, had a rodent ulcer, about one third of an inch in diameter, on the right temporal region. It had existed for two years, giving rise to annoyance by bleeding when rubbed with a towel. It was treated by ionisation with a zinc electrode, a current of 10 ma. being passed for ten minutes. In fourteen days a dry scab peeled off, and the ulcer had healed, and now, eleven months later, it remains healed.

**Case 2.**—A lady, aged about 55, had a rodent ulcer, $\frac{7}{16}$ in. in diameter. This caused similar trouble, owing to the bleeding, and had also existed about two years. It at once responded to treatment, and after two sittings, using a current strength of 12 ma. for twenty minutes, the ulcer had healed. But although the ulcer healed kindly, the hard raised edge did not disappear, and after five sittings at intervals of three weeks, it was still distinctly in evidence. I therefore attacked it with a zinc needle, puncturing it in four different places, with a current of 2 ma. running for two minutes. Two such sittings caused this thickening to disappear, and the face, now four months later, remains smooth.

**Case 3.**—An elderly man, with a rodent over the right malar bone. This patient was an alcoholic, and it was very difficult to get him to bear the pain of the treatment. I began with 3 ma. only, but by the fifth sitting I got him up to 15 ma. The ulcer was soundly healed, but the edge was not smoothed away when he discontinued attendance.

**Case 4.**—A male, aged 69, had had a rodent ulcer, $\frac{5}{16}$ in. by $\frac{1}{2}$ in., for some months. After one treatment, in, July 1906, the ulcer healed, and remained healed until the following December, when it broke down. Two doses of about 3 ma. for thirty minutes caused it to heal again, but after a month it again broke down, and eventually he went to my friend, Dr. Reginald Morton, who excised the ulcer, and treated the wound with X-radiation. It remains healed at the present time.

**Case 5.**—A male, aged 40 years, came with a rodent ulcer extending the length of the left lower eyelid. He was greatly distressed, as he had been told that he must have the whole eyelid cut away. Two applications of 7 ma. for ten minutes caused the ulcer to heal, and he is still under treatment to endeavour to remove the indurated edge.

**Case 6** is interesting in showing what zinc ions can do in an absolutely hopeless case. The man, whose photograph I show you, came under my care because he was suffering intense pain in the head. I found the pain was caused by the ravages of an enormous rodent ulcer, which had eaten away nearly all the concha of the ear and the side of the face, and involved his facial nerve, causing paralysis of that side of the face. I attacked it with zinc ions, more in the hope of cleaning the surface and reducing the horrible fœtor of it than anything else, but was very glad to find that the treatment relieved the pain, and enabled him to get up and move about again. He had for several weeks been confined to bed, and could only swallow liquids. I worked away for twelve weeks, giving a ten-minute sitting to some part of the ulcer each day, with the result that the diameter of the ulcer was diminished by one inch in each direction. After the first few applications a large part of the ulcer became so clean that I was able to get some grafts to live on it, his power of swallowing solids returned; his strength improved so that he could walk a mile. He returned to his home at Southampton, and I lost sight of him, but I heard that he died about three months later, probably, I think, from septic poisoning, which found entrance in what was left of the external meatus. I should like to insist that the time and trouble of the treatments were well repaid by the freedom from pain and the cleaning up of the ulcer which they occasioned.

**Case 7** is rather disappointing, inasmuch as it is not absolutely healed at the present moment, but is very instructive in showing how the disease can be kept in check, even when it cannot be entirely mastered. A lady, aged 44 years, first consulted me about a rodent ulcer of the right cheek, in November, 1901. She refused all operative interference, and it was treated by the static effleuve, by X-radiation, and by radium bromide until June, 1905, when, on Dr. Lewis Jones's advice, zinc ionisation was begun. Improvement at once showed itself, and the ulcer, which measured 41 by 20 millimetres was firmly healed after twenty-one sittings. This I reported at a meeting here in Bristol in August, 1906. Since then there have been small extensions of disease just where the funnel of the ear joins the face, the result of which has been to separate the lobe of the ear in rather an unsightly manner; but the disease is held in check, the face is free from pain, and the scar tissue covering the site of the large ulcer is wonderfully soft and smooth.

These are cases for which I think credit may be claimed for zinc ions; but before leaving the subject of rodent ulcer I should like to relate one other case—a man, aged about 70 years, who had an ulcer at the inner canthus of the right eye. It was treated with zinc on five occasions, without the slightest improvement. I then excised the ulcer, and the pathologist reported it to be a squamous epithelioma. It is interesting to see that although the ulcer looked just like a rodent, it was not rodent, and it did not respond at all to the zinc ions.

On two other occasions zinc ions have been of use to me.

A lad, suffering from granular lids, had been for some time under treatment by having them rubbed with mitigated stick, until it was hard to say whether the patient or the doctor was the more tired. One application of 1 ma. for two minutes caused such an improvement that he went to work, and states that his eyes now give him no trouble.

The second was an old lady, with a patch of lupus, about 2 in. by 4 in., on the back of the left hand and wrist, sent to me for X-ray treatment in July, 1907. She was so feeble that after four treatments she could no longer bear the walk to my house. I therefore attacked it on nine occasions with zinc ions. The effect was to clean up the ulcers and prevent the formation of fresh foci, and after long dressing with either a solution of zinc sulphate or an ointment of oleate of zinc healing of nearly the whole surface took place. Then the patient developed an acute nephritis and died. However, she practically died cured as far as her arm went.

As an instance of the use of salicylic ions, I may quote the case of a young woman, aged 34, who had, in December, 1907, an acute neuritis of her right brachial plexus, which subsequently involved the left plexus also. She was treated off and on until July, 1908, with the constant current, each application of which gave some days of ease and nights when sleep was possible. For a recurrence of pain, September and December, 1908, she was treated on eleven occasions with a static breeze. This, like the constant current, gave temporary relief, but did not effect a cure. So, in January of this year, I started to drive in salicylic ions, using a pad large enough to cover the plexus in the neck and a current of 20 ma. for half an hour. The first application gave most encouraging results, and after eight sittings she declared herself well. No drug treatment was associated with this.

One other case I should like to describe. It is of great interest to me, but, unfortunately, I am not able to record the absolute cure of the patient. She is a nurse, aged 27, who in July, 1908, complained of pain in the left ear. This pain was considered to be neuralgic until September, when a discharge was noticed, and an ulcer formed inside the pinna. In October the Lœffler bacillus was found in the discharge, and the patient spent four weeks in an isolation hospital, where the ulcer healed and she was discharged. A fortnight later the scar broke down and has remained unhealed ever since. She has been treated with large doses of antitoxin, and on two occasions the ulcer was scraped under an anæsthetic, and swabbed with a solution of lactic acid. It has also been treated with pyocyaneus and hydrogen peroxide. On April 15th my friend, Dr. James Pearse, asked me if I thought ionic medication would do any good. I got a report that the Lœffler bacillus was still found on cultivation from the discharge. On April 19th I gave an application of copper, using a current of 7 ma. for ten minutes. As the patient was frightened by the battery, this was as strong a current as could be borne. On April 22nd the crust which had formed became loose, and was easily lifted away. The surface of the ulcer looked clean, and the greater part of the edge appeared healing. On April 27th, as a crust had again formed, I gave a dose of 10 ma. for ten minutes. This again cleaned the ulcer, and healing went on. On May 8th the ulcer was divided into two by a band of epidermis across its floor, and as I thought, owing to its position inside the pinna, a bit might have escaped the copper ions, I bent out a cup-shaped electrode to fit more accurately the inside of the pinna, and gave another dose of 10 ma. for twelve minutes. To-day the ulcer has a crust over a good part of it, but it shows a healing edge where uncovered.

I have been much puzzled as to the best dressing to apply between the sittings. Obviously, a wet dressing kept on with a pad of wool and a bandage converts the ulcer into a first-class incubator, so I used wool which had been soaked in 2 per cent. copper sulphate and dried, packed into the pinna. A crust still formed, so I tried the effect of a wet dressing. The crust still formed. I should be glad of any suggestion as to the best way to dress the ear.

These are the cases which I wish to describe to you.

It remains to describe a little more fully the technique of the proceeding.

The requisite instruments are a constant current battery capable of driving a current of 30 to 40 ma. through the human body, a milliampère meter capable of reading the above current, and suitable electrodes and connecting wires.

Personally, I prefer my battery to be made up of Leclanché cells, as I find these are the easiest to clean up and replace in case anything goes wrong when far away from instrument makers. The milliampère meter I prefer is the variety in which the needle floats in spirit, as this saves all troublesome levelling of the instrument or battery. The electrodes must be cut to shape, so as to just overlap the edges of the surface to be treated. The larger the indifferent electrode the better, but I find a cylinder of sheet zinc one inch in diameter and covered with four thicknesses of flannel very useful, as the patient can hold it in a basin of water, thus preventing the annoying drying which so often takes place when a pad on the back is used.

We will suppose that a rodent ulcer of the face is to be treated. The patient is placed lying on a couch, with the head supported so that there can be no jump when the current is switched on. Any greasy application which may have been put on the ulcer and surrounding skin is carefully removed. A suitably sized electrode of zinc is selected and carefully polished. I find the best way to arrange my electrodes is to have a holder such as I show here, made from a bit of brass tube of a size just sufficient to allow a No. 7 brass screw to slip into it and be firmly held. To the head of this screw a bit of ordinary roofing zinc, which can be easily bent to shape with the fingers and cut to size with ordinary scissors, is soldered. Thus, at practically no expense any number of electrodes can be quickly made.

A pad formed of at least sixteen thicknesses of lint is then soaked in a 2 per cent. solution of zinc sulphate, and laid over the ulcer; the polished electrode is placed and held on this with gentle pressure, after having been connected with the positive pole of the battery. It is well to test the poles of the battery pretty often, as the shifting of a current reversing in dusting is easily overlooked. A bit of wet blue litmus paper is reddened at the positive pole.

The patient grasps the pad connected to the negative pole, and the current is slowly switched on. It is now necessary to pay attention to the dial collector, and see that the collecting arm works smoothly and touches the record stud before it quite leaves the first, so that the rise of current takes place without any jerk. For the same reason it is very essential to see that all connections of battery cords and electrodes are quite firm. Professor Leduc insists that all connections should be soldered.

The absolute strength of the current must be fixed by what the patient can bear, but may be taken to be 2 or 3 ma. per square centimeter of surface.

The length of the sitting is also fixed by the tolerance of the patient; but supposing the current to be about 3 ma. per square centimeter, ten minutes to a quarter of an hour will probably suffice.

It must be borne in mind that the quantity of zinc driven in, and the depth to which it is driven, will vary directly with the time and current strength.

After an application I leave the pad which has been used during treatment as a dressing, partly to avoid disturbance and the providing of another dressing, and partly with the idea that there may still be some charged ions left in it.

If the ulcer is looked at after the treatment the surface will be seen to have turned a pearly white, while the skin around is reddening. This reaction passes off in about forty-eight hours, and the ulcer looks clean and takes on a healing action. I find it best to allow three weeks between treatments, and to use a simple dressing, such as lotio rubra, during the interval.

When breaking the current it is necessary to evenly and gradually reduce the strength, else painful shocks will be caused.

I would repeat that the electrode must be polished quite brightly before each application, and I find this is best done with a bit of the finest glass-paper used by cabinet makers.

Dr. Lewis Jones said it gave him great pleasure to speak on the subject of ionic medication, because he might claim to be the godfather of the method in this country, and the method was one well worthy of attention.

He wished, in the first place, to congratulate Dr. Herbert Tayler on his excellent paper, and as that paper had been mainly concerned with the clinical aspect of ionic medication, he (Dr. Jones) would like to say something of its scientific side.

Dr. Lewis Jones then arranged an experiment to demonstrate the migration of ions under the influence of the electric current. A circuit was arranged through a number of layers of parchment paper, formed by folding a long strip upon itself many times; they were moistened with a dilute solution of sulphate of soda, and also contained a trace of phenolphthalein, and were placed between copper electrodes.

Under these circumstances the passage of the current would cause a movement of ions of copper into the paper from the positive pole, the source of these copper ions being the metal of the positive electrode, so that if copper were subsequently found in the pores of the paper it must have been derived from the electrode, and carried into the parchment paper with the current.

The current was started at 14 milliampères, and allowed to flow for twenty-five minutes. The strips of paper were then unfolded, and on the side of the positive electrode the copper was shown to have penetrated to a considerable distance by treatment with potassium ferrocyanide, which gave the brown colour characteristic of the presence of copper. At the negative pole no copper had entered the paper, but there was a purple stain due to the interaction of the phenolphthalein with ions of hydroxyl, which had penetrated inwards through many layers of the paper, starting from the negative pole. The ionic movements set up in the experiment were, therefore, entirely different at the two poles.

The principles of ionic medication depended upon this influence of a current in producing a penetration of the ions of active drugs into the skin and subjacent tissues.

The effects of ionic medication were superior to those obtainable by means of lotions and ointments, by reason of this penetration, for these externally-applied lotions, etc., penetrated only very slightly, and if there were germs growing within the substance of the cells these were practically untouched by ordinary external applications ; but with the aid of electricity such intracellular medication could be obtained, and might make considerable difference in the result, and there were many disorders in which the power to introduce a drug by ionisation might be of great value, so that in ionic medication they had a new weapon. It remained to find out the fields of action in which ionic medication could be most usefully applied.

In the choice of chemical bodies for ionic medication one must know something of the ionic properties. Thus, chlorine as chlorine water was a strong bleaching agent, but chlorine ions have not this property. The chlorine ion, therefore, could not be used for oxidising and disinfecting purposes, as these were properties of the concentrated chemical, but not of its ions, for chlorine ions were abundant in normal saline solution, or in the juices of the body. Strychnine, on the other hand, had definite ionic properties, and was an ion capable of introduction into the body by means of a current, and of producing its well-known physiological actions. Carbolic acid was an alcohol, and did not dissociate into ions when dissolved in water. All neutral salts which were soluble in water formed ions when dissolved, and the same was true of acids and of alkalies. Examples of ions useful in medicine are those of zinc, copper, magnesium, quinine, cocaine, strychnine and salicylic acid. The properties of many other ions remain to be discovered. Only a few have yet been handled, and those mentioned have been found useful.

In the skin the ions enter quite well for a little distance, but when they reach the interior of the body some undergo chemical changes which put them out of action. Nearly all the heavy metals form insoluble phosphates, and are deposited as such in the tissues, and penetrate no farther with the current.

In the case of zinc, this has a slight advantage over the other heavy metals, in that its phosphates are not entirely insoluble. Mercury and silver are metals which do not readily lend themselves to ionic medication. Silver ions are put out of action when they meet chlorine ions. Thus silver is only useful for the most superficial affections, as it is precipitated almost at once as a chloride. If mercury ions are used, gold electrodes amalgamated with mercury must be employed as the source of the mercury.

Practically all metals must be introduced from the positive pole.

Even if ionic medication were suitable only for quite superficial purposes, there would yet be a considerable field for its application. It has been found useful for skin affections, such as lupus, lupus erythematosus, rodent ulcer, septic and tuberculous ulceration, eczema, and similar affections.

As lupus is one of the most troublesome of diseases, any method which would enable one to treat lupus successfully would be of the highest value. In the treatment of lupus the results had been very encouraging, and zinc, aniline, or copper should be tried. In an obstinate case of tuberculous ulcer on the inside of the mouth success had been obtained, but only after repeated applications, and copper was found more efficacious than zinc.

Rodent ulcer had yielded well to ionic treatment, and numbers of cases had been successfully treated by many different observers. When taken in time rodent ulcer was readily cured by zinc ions, but the extensive ulcers sometimes met with were not suitable for ionic treatment because of the pain associated with the applications over a large area.

Success has also been reported in the treatment of certain intractable rectal ulceration by ions, and also in vaginal and uterine disorders.

In Mooren's (so-called rodent) ulcer of the eye, which was a most intractable complaint, the usual treatment consisted of cauterisation, but in two cases treated by ionic medication one was completely cured after a single application of zinc ions, and the other after two or three. Granular lids and other affections of the conjunctiva also deserved trial with ions.

In the treatment of sinuses it was difficult to ensure the penetration of the ions into every part of the affected area. It was useless to treat a portion only of a suppurating sinus, and for this reason it was by no means easy to cause a sinus to heal by ionisation, unless one were lucky enough to reach every part of its interior. The effect was only temporary where it was not done completely, as re-infection occurred.

Dr. Lewis Jones congratulated Dr. Tayler on the case of neuritis which he had relieved by ions. Trigeminal neuralgia of severe type was sometimes benefitted markedly by ionic medication of the skin of the face, using salicylic or quinine ions. It was possible that certain ions introduced into the skin of the face might travel along the lymphatics of the fifth nerve to the Gasserian ganglion, and thus produce beneficial results when the neuralgia was the result of changes there.

The solutions to be used were, as a rule, to be of a strength of

1 per cent. Metals, alkalies, alkaloids were driven in from the positive pole, acids from the negative. Neutral salts formed the best material for the solutions; thus quinine was best introduced from a solution of its hydrochlorate. Salicylic acid from a solution of sodium salicylate, and so on. In the case of zinc and copper, the positive electrode should be of that metal, and the solutions should be sulphates or chlorides of the metal. Where the corresponding metal could not be used for the electrode, a carbon or platinum electrode should be employed, and care should be taken to have abundance of the solution in numerous layers of lint. For the introduction of ions from the negative pole the same precautions should be taken, but any ordinary metallic electrode might be used.

*Discussion.*

Dr. J.W. McBain said that on the physico-chemical side of ions a great deal of work had been accomplished, and he foresaw a great field of usefulness for it in medical practice. In a large number of cases the salts were diffused through the membranes of the body. In those cases where it would be impossible to get a metal electrode in contact with the surface, it might be possible to explore with a glass tube filled with the solution. In applying metallic or alkaline radicles the positive terminal should be employed, while for all acid radicles it would be necessary to reverse the current.

Dr. W. Kenneth Wills said he had had no more striking results of ionic treatment than in rodent ulcer, and he had hoped to show a patient under treatment with zinc ions. She was treated about eighteen months ago with one application, but at the time only half the disease was done, and she was prevented from continuing the treatment. Later the patient came up because of the increasing size of the untreated part. The part that had been subjected to ionic medication was well, except for a small nodule in the centre; whilst the other half, freed by the part that had healed, had fulminated, and had grown very considerably. He inquired what had given Dr. Jones the best results in the treatment of lupus erythematosus, as zinc had been found to be of little use. Not much success had been gained in treating ulcers where cartilage and bone were involved. Several interesting cases of sinuses had yielded well to iodine ions. He was not prepared to say that the ion mattered in these cases, although the ion made the sinus sterile for a considerable time.

Mr. J. Taylor mentioned the benefit obtained in ozœna by the use of magnesium and copper ions, and mentioned a case where a child's deafness had been incidentally improved while undergoing ionisation treatment for another complaint. He inquired as to benefit derived from ions in ringworm treatment.

Dr. G. Parker said he was specially interested in the fact that it was possible to introduce single ions, though their properties were yet to be learnt. In giving cataphoresis he inquired if the electrodes should be placed close together, or at some distance away from each other. He questioned whether a better result would not be obtained by cutting off the circulation as much as possible, such as by using a rubber ring on a gouty finger, or by stopping the circulation by using one of Bier's bandages. He thought that care should be taken that the battery did not polarise; and he presumed the question of reversal of current was not necessary.

Dr. Hey Groves asked for more information as to the treatment of malignant disease by ionisation, and what advantages, if any, it possessed over the radical excision of the growth.

Dr. Roxburgh stated a case of a man operated on for fistula in ano, which had obstinately refused to heal under surgical treatment, but zinc ions had been used with great success, and after four applications the wound was only half the length it was at the beginning.

Dr. Watson Williams asked as to Dr. Jones's success with diphtheritic ulcers in the latent form, and if there was any disadvantage in using the strongest possible solutions. He had used magnesium ions with a current of 25 or more milliampères for thirty minutes, and had found this useful. With regard to the word "sinus," he asked if Dr. Jones referred to the accessory sinuses of the nose.

Dr. Vickery said that three years ago he had used ionisation, but possibly on the wrong case. It was one of superficial epithelioma of the hand. The treatment gave great pain to the patient, and after three applications refused to continue the treatment. He referred to the nature of the resulting covering of the ulcer—similar to the white of an egg, or as if it had been daubed with strong carbolic.

Dr. Bowker gave his experience with chlorine ions, which had had great influence in a case of Dupuytren's contraction, for which he had given chlorine in a 2 per cent. solution in 50 to 80 milliampères for an hour at a time. The patient had to leave at the end of a week, but he was able to extend the finger. He asked if Dr. Jones had any remarks or suggestions to make as to the best form of lithium salts in arthritic affections. A patient suffering from gout had lithium between the joints, and chlorine in the other joints, and by these means warded off for a month all gouty symptoms. He inquired if any hydroxyl ions were useful in stricture of the urethra, and what strength of current one was justified in using.

Dr. NIXON inquired whether the single prolonged application had any advantage over repeated short ones ; also whether the distribution of ions into the wall of a cavity packed with gauze moistened with the ionisable solution took place fairly evenly. He described some results of treatment of papillomata and warts, saying that the naked electrode had advantages over the lint pad upon small areas. He also asked for information as to ionic treatment in fissure and pruritus ani.

The PRESIDENT thanked Dr. Tayler and Dr. Lewis Jones for their kindness in opening the discussion, and congratulated the members on the instructive discussion. He was sorry to hear it hinted that the ion used made no difference. They knew that to a certain extent electricity suffered from the vague way in which it has been handled, and they ought to know exactly with what they were dealing. When salicylic ions entered the body he thought they should be detected in the urine. He inquired as to the length of time and results in the treatment of neuralgia, and what ions had been used.

Dr. LEWIS JONES, in reply to questions, said that for the ionisation treatment of neuralgia, quinine and salicylic acid had been found valuable, and of the two he preferred salicylic acid, as the ions were well tolerated by the skin. Long sittings of from twenty to twenty-five minutes were necessary, and currents of twenty or thirty milliampères.

He thought that fissure of the anus and pruritus ani were suitable subjects for trial by ionic treatment, although personally he had had no experience of their treatment by the method.

As regards excision versus ionisation of rodent ulcer, doubtless much might be said on both sides ; but excision left a scar, and there was a risk of re-infection of the wound during the operation, and consequent recurrence.

He thought that the direct application of a bare metal electrode without any layers of moistened lint between it and the skin, as described by one of the speakers, would be more painful.

To treat ringworm by ionisation was a difficult matter, as it was impossible to secure the penetration of the ions to all the hair follicles, and it was useless to leave any still containing the fungus, as recurrence was certain to take place. By using zinc or copper ions he had been able to produce a growth of healthy hair on patches affected by ringworm, but the ringworm fungus could still be discovered lurking in a few follicles.

In the case of Dupuytren's contraction referred to by a speaker, he considered that if more time had been given the result would have been better.

When he used the word "sinus," he meant a suppurating sinus remaining after an operation wound, and not the anatomical sinuses of the nose.

In lupus erythematosus the ions of zinc and of copper had given good results, although success had not followed in all instances.

---

